# Cardiogenic shock in a patient with hypothyroid myopathy responsive only to thyroxin replacement: a case report

**DOI:** 10.1186/1757-1626-3-66

**Published:** 2010-02-23

**Authors:** Sanath Dharmasena, Olga Burzyantseva, Suriya Jayawardana, Vijay A Rupanagudy, Krishnan Pathmanathan

**Affiliations:** 1 Department of Medicine, Coney Island Hospital, 2601 Ocean Parkway, Brooklyn, NY 11235, USA; 2 Department of Cardiology, Coney Island Hospital, 2601 Ocean Parkway, Brooklyn, NY 11235, USA; 3 Department of Pulmonology, Coney Island Hospital, 2601 Ocean Parkway, Brooklyn, NY 11235, USA

## Abstract

The effect of hypothyroidism on the cardiovascular system has been well documented. Cardiac dysfunction due to hypothyroidism manifests as both systolic and diastolic dysfunction of the heart leading to cardiac arrhythmia and congestive heart failure. Its presentation in the form of refractory hypotension is rare. We describe a 52 year old man on whom Hypothyroid Cardiomyopathy manifested as cardiogenic shock responsive only to thyroxin replacement.

## Case presentation

A 52 year old man with nausea and vomiting of two days duration was found to be hypotensive and in acute hypoxic respiratory failure requiring mechanical ventilation. The patient is known to have hypothyroidism and he has been non compliant with oral Synthroid therapy. Hypotension persisted in spite of aggressive initial fluid resuscitation. The electrolytes, renal function, white blood count and hemoglobin were all within normal limits. Blood TSH level was 69.45 mU/L (0.35-5.50 mU/L); T4 level was 0.454 ng/dl (0.93-1.7 ng/dL) and T3 level was 29.87 ng/dl (84-202 ng/dL) all indicative of severe primary hypothyroidism. The Chest x-ray revealed normal heart size with pulmonary congestion. The EKG revealed a heart rate of 96/min with normal axis and rhythm and without ischemic changes. Patient remained hypotensive in spite of initiation of dopamine infusion. A bed side echocardiography revealed a left ventricular ejection fraction (LVEF) of 30% with global hypokinesia. The isovolumetric contraction time and isovolumetric relaxation time were prolonged with increased myocardial performance index (MPI) of (0.56) indicating that there was both systolic and diastolic dysfunction. An echocardiogram done six month ago showed a normal LVEF and normal MPI.

Hypotension persisted in spite of high dose dopamine infusion. In the absence of evidence of myocardial ischemia hypothyroidism was considered to be responsible for the observed acute refractory hypotension/cardiogenic shock. Intravenous thyroxin at a dose of 0.050 mg was administered and the dose increased to 0.075 mg in the next 48 hours resulting in stabilization of the blood pressure and weaning off the dopamine and subsequently weaned off ventilator during the next 72 hours. A repeat echocardiography done in one month revealed normal LV function with LVEF of 60% with normal MPI of 0.35.

## Discussion

The effects of thyroid hormone on cardiac function are well documented [1]. Thyroid hormone has widespread effects on cardiovascular function [2]. Triiodothyronine, the active form of thyroid hormone, enters cardiomyocyte nuclei and modulates calcium flux, beta-adrenergic-receptor function, and the transcription of various contractile proteins. Both hyper and hypothyroidism can cause changes in cardiac contraction, In addition, severe hypothyroidism is associated with ventricular dysrhythmias, including torsades de pointes [3]. Hypothyroidism is associated with depressed chronotropy, increased peripheral vascular resistance, decreased cardiac contractility and decreased cardiac output and these effects are reversed with counteracting the hypothyroid state [4-6]. While cardiac dysfunction is well documented secondary to hypothyroidism, hypotension is rare and when it occurs it is resistant to conventional therapy in the form of intravenous fluids and vasopressors [7]. Hypothyroidism causes muscle relaxation and the isovolumetric relaxation and contraction time becomes prolonged and MPI increases indicating systolic and diastolic dysfunction [8]. Only when Cardiac dysfunction is severe, cardiac out put can be decreased enough to cause cardiogenic shock. In such situations urgent thyroxin replacement has been shown to improve cardiac function and restore hemodynamic stability [9,10] as was the case in the patient we described.

## Conclusion

Hypothyroid cardiomyopathy can manifest as cardiogenic shock and only thyroxin replacement can restore hemodynamic stability by reversing the systolic and diastolic dysfunction.

## List of Abbreviations

(LVEF): left ventricular ejection fraction; (MPI): myocardial performance index.

## Consent

A written informed consent was obtained from the patient for publication of this case report. A copy of the written consent will be made available on request.

## Competing interests

The above mentioned authors declare that they have no competing interests.

## Authors' contributions

DS, OB, SJ treated the patent and were responsible for writing the paper and looking up the background references. RV, PK was responsible for over all coordination and final proof reading. All the above mentioned authors read and approved the final manuscript.
